# Design and evaluation of a computed tomography (CT)-compatible needle insertion device using an electromagnetic tracking system and CT images

**DOI:** 10.1007/s11548-015-1176-3

**Published:** 2015-04-07

**Authors:** Navid Shahriari, Edsko Hekman, Matthijs Oudkerk, Sarthak Misra

**Affiliations:** 1 Center for Medical Imaging - North East Netherlands, University of Groningen, University Medical Center Groningen, Hanzeplein 1, 9713 GB Groningen, The Netherlands; 2 Department of Biomechanical Engineering (MIRA-Institute for Biomedical Technology and Technical Medicine), University of Twente, Drienerlolaan 5, 7522 NB Enschede, The Netherlands; 3 Department of Biomedical Engineering and Center for Medical Imaging - North East Netherlands, University of Groningen, University Medical Center Groningen, Hanzeplein 1, 9713 GB Groningen, The Netherlands

**Keywords:** CT-guided procedures, Computer-assisted surgery, Medical robots and systems, Image-guided control, Electromagnetic tracking, Minimally invasive surgery

## Abstract

**Purpose:**

Percutaneous needle insertion procedures are commonly used for diagnostic and therapeutic purposes. Although current technology allows accurate localization of lesions, they cannot yet be precisely targeted. Lung cancer is the most common cause of cancer-related death, and early detection reduces the mortality rate. Therefore, suspicious lesions are tested for diagnosis by performing needle biopsy.

**Methods:**

In this paper, we have presented a novel computed tomography (CT)-compatible needle insertion device (NID). The NID is used to steer a flexible needle (${\phi }0.55\,\hbox {mm}$) with a bevel at the tip in biological tissue. CT images and an electromagnetic (EM) tracking system are used in two separate scenarios to track the needle tip in three-dimensional space during the procedure. Our system uses a control algorithm to steer the needle through a combination of insertion and minimal number of rotations.

**Results:**

Noise analysis of CT images has demonstrated the compatibility of the device. The results for three experimental cases (case 1: open-loop control, case 2: closed-loop control using EM tracking system and case 3: closed-loop control using CT images) are presented. Each experimental case is performed five times, and average targeting errors are $2.86\pm 1.14$, $1.11\pm 0.14$ and $1.94\pm 0.63\,\hbox {mm}$ for case 1, case 2 and case 3, respectively.

**Conclusions:**

The achieved results show that our device is CT-compatible and it is able to steer a bevel-tipped needle toward a target. We are able to use intermittent CT images and EM tracking data to control the needle path in a closed-loop manner. These results are promising and suggest that it is possible to accurately target the lesions in real clinical procedures in the future.

## Introduction

Percutaneous needle insertion into soft tissue is a common minimally invasive surgical procedure. Clinical needle procedures are used for diagnostic and therapeutic purposes such as biopsy, brachytherapy and ablation. These procedures are commonly performed manually by clinicians. Different imaging modalities, such as computed tomography (CT), magnetic resonance imaging (MRI) and ultrasound, are used to provide feedback to the surgeon to reach the target accurately. Although accurate localization of lesions is possible using current imaging technology, they cannot yet be precisely targeted [1]. Cancer-related diagnoses and therapies of the lung are among the important topics in the field of percutaneous procedures. This is due to the high mortality rate of lung cancer worldwide (1.59 million deaths in 2012) [2], and also risk of complications such as hemothorax and pneumothorax [3]. Early detection can increase the chance of survival [4].

Due to importance of early detection, usually a needle biopsy is performed when a suspicious lesion is observed in CT images. The tissue is then tested for diagnosis. The procedure begins with a CT scan of the region of interest. The clinician determines the insertion point using a radio-opaque grid and laser alignment system of the CT scanner. The biopsy needle is then inserted for several millimeters into the chest. The insertion angle is checked several times during the procedure by performing new CT scans. If the needle is in the correct direction, the clinician further inserts the needle, otherwise the needle is retracted and re-inserted until the needle is properly aligned. Finally, the biopsy is taken when the needle is close enough to the lesion. Each time a new CT scan is taken, the clinician must leave the CT room. This causes delay in the procedure, and it is not convenient for clinicians. The number of attempts (re-positioning the needle) to reach the lesion depends on the clinician’s experience and lesion position. Near-real-time imaging of the lesion using CT fluoroscopy (CTF) is possible to reduce the number of attempts. It was shown that the success rate is improved while using CTF [5]. The risk of complications increases with the number of insertion attempts [3].

### Related work

Different robotic setups have been developed to perform needle insertion procedures aiming at increasing targeting accuracy and thereby minimizing the number of insertion attempts [1, 6]. In this work, we are specifically interested in using needle steering to address the mentioned problems, which will be briefly discussed along with different robotic setups.

#### Needle steering methods

Different steering methods have been proposed in the literature. Needles with a symmetric tip can be steered by moving the base of the needle [7]. On the other hand, needles with an asymmetric tip (bevel-tipped) [8], a pre-bend/-curved tip [9] or an actuated tip [10] deflect due to the tip shape.

**Fig. 1:**
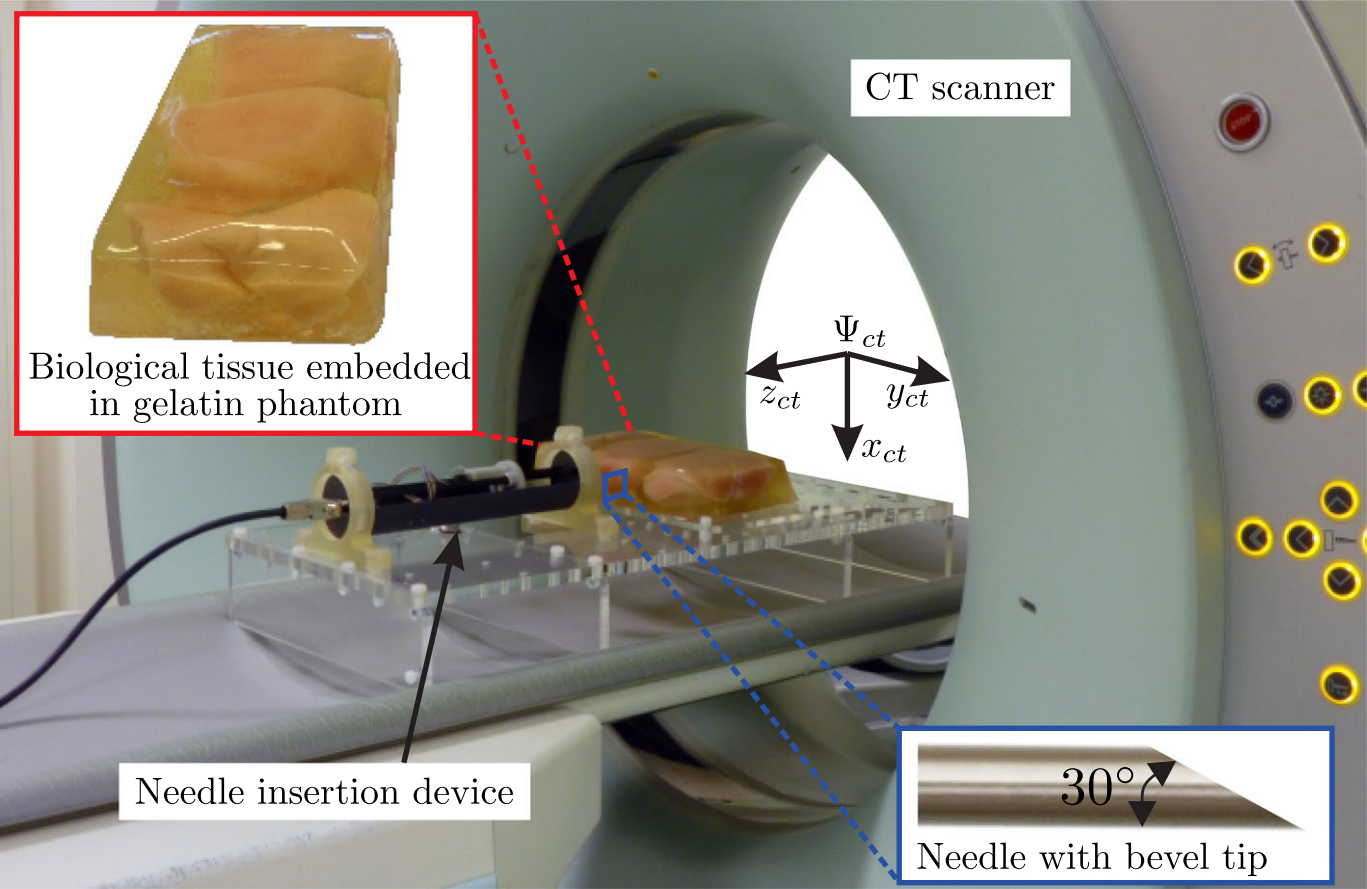
The experimental setup used for steering a bevel-tipped needle. The needle is steered toward a virtual target in biological tissue embedded in a gelatin phantom using computed tomography (CT) images. The *top* *inset* shows the phantom, and the *lower* *inset* shows the needle with a bevel at the tip. The frame ($\varvec{{\varPsi }}_{\text {ct}}$) represents the CT scanner coordinate system

Needles used for clinical procedures such as biopsies and ablations usually have an asymmetric tip. Tissue surrounding the needle and the force required to cut the tissue cause interaction forces at the needle. In the case of bevel-tipped needles (Fig. 1, lower inset), the forces which are applied to the tip result in transverse load [11]. This causes needle deflection during the insertion. The needle deflection can be used to steer the needle along a non-straight path toward a target in the tissue. The needle trajectory can be controlled to follow a pre-defined path by modeling the deflection. The deflection can be modeled based on the kinematics of the needle [12] or based on mechanics of needle–tissue interaction [11]. The amount of deflection depends on several parameters, such as bevel angle, insertion speed, needle diameter and tissue stiffness. Webster et al. [12] modeled motion of bevel-tipped needles as a unicycle and bicycle, where they assumed the needle describes a path of constant curvature. Other researchers showed that the curvature can be controlled through duty-cycled spinning of the needle [13, 14]. Abayazid et al. [15] developed a three-dimensional (3D) steering algorithm which minimizes the number of needle rotations. The control loop, for the mentioned steering algorithms, can be closed using feedback from the needle position. Ultrasound [16], MRI [17] and CT [18] images are used to track the needle in tissue. Fiber Bragg grating sensors and electromagnetic (EM) tracking sensors are also used for needle tracking [15, 19].

#### CT-compatible devices

Different CT-compatible robotic setups have been developed to help clinicians better target lesions. These robotic setups can be categorized based on their insertion principle and structure.

Considering the insertion principle, it is possible to divide these setups into positioning devices and needle insertion devices (NID). Examples of positioning devices can be found in the literature [6–20]. Such systems only position and orient a needle holder, and the insertion is done by the clinician. The optimal position and orientation to insert the needle are determined using diagnostic images. The needle holder is then positioned and oriented accordingly, and the needle is inserted manually. On the other hand, NIDs both position and orient the needle and also insert the needle into the tissue. The insertion could be fully-automated [3, 18], or it could be semi-automated [21]. In the fully-automated control, the needle is inserted considering the relative positions of the target with respect to the needle tip. However, in semi-automated control, the clinician is in the loop during the procedure [21]. None of the existing CT-compatible setups provide needle rotation about its axis, which is useful for needle steering.

It is also possible to classify the mentioned setups based on their structure. The device could be patient-mounted [1] and table-mounted [22], or it can have a base on the ground [6]. This categorization is important because one of the issues in needle insertion in thorax and abdomen is that the body moves due to respiration. The patient-mounted devices compensate for the body motion passively because they move with the patient [1]. On the other hand, table-mounted and ground-mounted devices require an online tracking system to compensate for patient motion [18]. The tracking data are then used to compensate for the body motion in the robot control algorithm. Another advantage of patient-mounted devices over the other two is that they are usually smaller, lightweight and provide better access to the patient for the clinician.

### Contributions

In this work, we present a novel CT-compatible NID which is capable of rotating the needle while inserting it into the tissue. The compatibility of the device is demonstrated via noise analysis of CT images. The NID has been used to steer a bevel-tipped needle in a phantom with biological tissue toward a virtual target. EM tracking and CT images are used in two separate experimental cases as feedback to the steering algorithm, and the results are compared. To the best of our knowledge, this is the first CT-compatible NID which is capable of steering needles through a combination of insertion and rotation.

The paper is organized as follows: In “Design” section, our CT-compatible NID design is discussed. The experimental setup, plan and results are presented in “Experiments” section followed by conclusion and directions for future work in “Discussion” section.

## Design

**Fig. 2:**
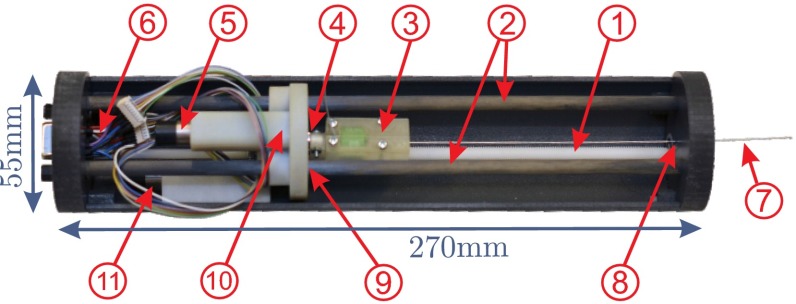
Prototype of computed tomography-compatible needle insertion device: ① Drive shaft. ② Guide bars. ③ Needle gripper. ④ Ball bearing. ⑤ Motor for needle rotation. ⑥ Cables to the low-level controller. ⑦ Needle. ⑧ Insertion point. ⑨ Bushing. ⑩ Carriage. Motor for insertion/retraction

In this section, the design of a CT-compatible NID is explained. Considering the discussion in the previous section, we are using bevel-tipped needles to perform needle steering. At least two degrees of freedom (DOF) (insertion and rotation) are needed to control the needle trajectory. Current CT scanners [such as Siemens Somatom Sensation 64 (Siemens AG, Munich, Germany) and Brilliance CT (Philips Healthcare, Best, The Netherlands)] have a gantry opening of about 820 mm. There is approximately 300 mm free space around the abdomen to place the device while a patient is inside the bore. As depicted in Fig. 2, the designed NID is a cylinder of 55 mm in diameter and 270 mm in length; 150-mm-long needle is used in the device and the maximum insertion length is 120 mm. The device is designed such that the insertion point (Fig. 2, ⑧) and all metallic parts (such as motors and electric connections) be placed at two opposite sides of the device. This helps to minimize the noise and artifacts in the CT images as much as possible.

The needle is placed in a gripper which is attached to the carriage using ball bearings. The carriage is moved forward (insertion) and backward (retraction) using the drive shaft. The drive shaft and the carriage have external and internal ISO metric screw threads. The carriage slides on two carbon fiber tubes. Since the force applied to the carriage from the drive shaft is not symmetrically distributed, friction acts at the contact points of the carriage and the guiding carbon tubes. Therefore, oil-free bushings are used to achieve a smooth motion (Fig. 2, ⑨). The insertion and rotation are controlled using two motors. The motors are brushed-DC 1016N012G with a HEM-3 quadrature encoder and a 10/1 planetary gearhead of 1:4 ratio (Faulhaber Group, Schnaich, Germany). Spur gears with transmission ratio of 1:3 are used to transmit the motor torque to the drive shaft and needle gripper. The body is 3D printed using acrylonitrile butadiene styrene (ABS), and the shaft is made of polyoxymethylene (POM). Ball bearings with plastic inner and outer races with glass balls are used in places that may interfere with CT images.

The low-level motor controller is a proportional-integral-derivative (PID) controller which is implemented on a ATMEGA328 (Atmel Corporation, California, USA) microcontroller. The motor speed is controlled through pulse width modulation (PWM) using the feedback from the motors encoders.

The high-level controller is based on the steering algorithm which is discussed in the following section. The motor set points are sent to the low-level controller using universal asynchronous receiver/transmitter (UART). The low-level controller then controls the motors to reach the set point.

## Experiments

In this section, first, the different components and parameters of the experimental cases are introduced. The experimental plan consisting of a CT-compatibility test of the device, and three steering cases are then explained. Finally, the results are presented and discussed.

### Setup

**Fig. 3:**
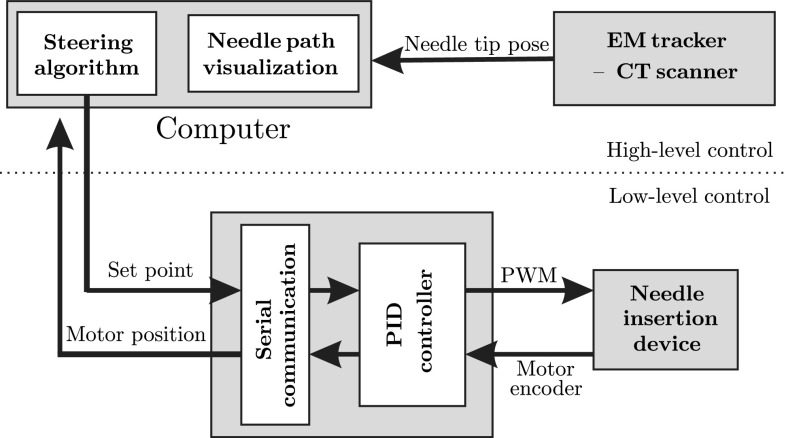
Block diagram of the experimental setup: The needle pose is measured using electromagnetic (EM) tracker or computed tomography (CT) images. The steering algorithm computes the amount of needle rotation needed. The control command (motor set point) is sent to the low-level controller. The low-level controller controls the motors using PID controller through pulse width modulation (PWM)

The experimental setup consists of the NID, low-level controller electronics, CT scanner or EM tracker and a computer. The block diagram of the experimental setup is presented in Fig. 3. Two different systems are used in the experiments to provide feedback to the needle steering algorithm. In one scenario, needle pose is calculated using CT images, and in the other scenario an EM tracker system is employed. The NID and the low-level controller are discussed in the previous section. The details about the CT scanner and the EM tracker system are provided here.

The CT scanner used in the experiments is the Siemens Somatom Sensation 64 (Siemens AG, Munich , Germany). The settings are the defaults used for abdomen scan, which are a tube voltage of 120 KVP, tube current of 409 mAs, pixel spacing of 0.6719 mm, slice thickness of 2 mm with 1.5 mm overlap and convolution kernel of B30f.

**Fig. 4:**
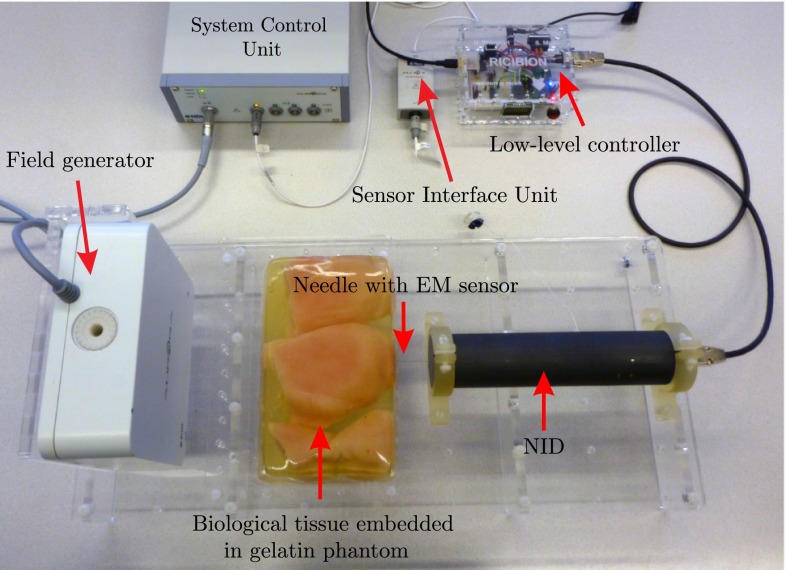
Experimental setup using Aurora electromagnetic (EM) tracker: the tracking system consists of a planar field generator, a system control unit, a sensor interface unit and a sensor embedded in the needle close to the tip. The system is able to track the sensor in a $500\,\times \,500\,\times \,500\,\hbox {mm}$ cube volume. The needle insertion device (NID) is controlled by the low-level controller to steer the needle in biological tissue embedded in gelatin phantom

A 5DOF EM sensor is embedded in a 0.55 mm needle to track the needle tip with the EM tracking system. This sensor is chosen due to its smaller size ($\phi 0.5\,\hbox {mm}$) with respect to 6DOF sensor ($\phi 0.8\,\hbox {mm}$). Aurora v2 EM tracker (Northern Digital Inc., Waterloo, Canada) is used for measuring the sensor pose 40 times per second [23]. The 3D position, pitch and yaw angles are measured by the system. The roll angle (rotation about needle axis) cannot be measured from the EM sensor, and therefore, it is calculated from the motor encoder. The assumption is that the torsion about the needle axis will cause only minimal offset between the tip and base angles. As depicted in Fig. 4, the EM tracking system consists of a field generator, a system control unit and a sensor interface unit. According to the manufacturer, the root mean square (RMS) of the position error is 0.7 mm and it is $0.20^{\circ }$ for the orientations, when the planar field generator is used.

### Plan

Two experimental scenarios are planned to validate the CT-compatibility and functionality of the device. The experimental plan is described in this section.

#### CT image noise analysis

The CT-compatibility of the device has been proved through noise analysis of CT images. It is discussed in literature that signal-to-noise ratio (SNR) is a fundamental concept in noise analysis. However, it does not characterize the noise completely [24]. One of the characteristics that is missing in SNR is the so-called noise texture. Noise texture is related to the spatial frequency distribution of the noise. Therefore, the noise power spectrum (NPS) is commonly used for analysis of CT images. NPS characterizes the noise texture by describing the noise variance as a function of spatial frequency. In other words, the NPS is the Fourier transform of the autocorrelation function and is computed as

1 $$\begin{aligned} \text {NPS}(f_x,f_y) = \frac{1}{N} \sum \limits _{i=1}^N \left| \text {DFT}_\text {2D}\left[ I_i(x,y)-\bar{I_i} \right] \right| ^2\frac{{\Delta }_x {\Delta }_y}{N_x N_y} \end{aligned}$$

where $f_x$ and $f_y$ are the spatial frequencies in $x$ and $y$ direction (Fig. 1), respectively. $\text {DFT}_\text {2D}$ is the 2D discrete Fourier transform, $I_i(x,y)$ is the signal in $i\hbox {th}$ region of interest (ROI), and $\bar{I_i}$ is the mean of $I_i(x,y)$. $N$ is the number of ROIs, and $N_x$ and $N_y$ are number of pixels, and ${\Delta }_x$ and ${\Delta }_y$ are the pixel spacing in $x$ and $y$ direction, respectively.

The NPS is computed using a homogeneous cylindrical phantom (e.g., water or plastic). The phantom is scanned, and several ROIs are sampled in a CT image. The Fourier transform is computed for each ROI and then averaged over all the samples, and the mean 2D NPS is calculated. It is also possible to collapse the 2D NPS to 1D by radially averaging the 2D NPS [24]. CT images are taken when the water phantom is in the CT bore alone and also when the NID is on top of the phantom to check the CT-compatibility of the device. 1D NPS is used to compare the resulting CT images.

#### Needle steering

**Fig. 5:**
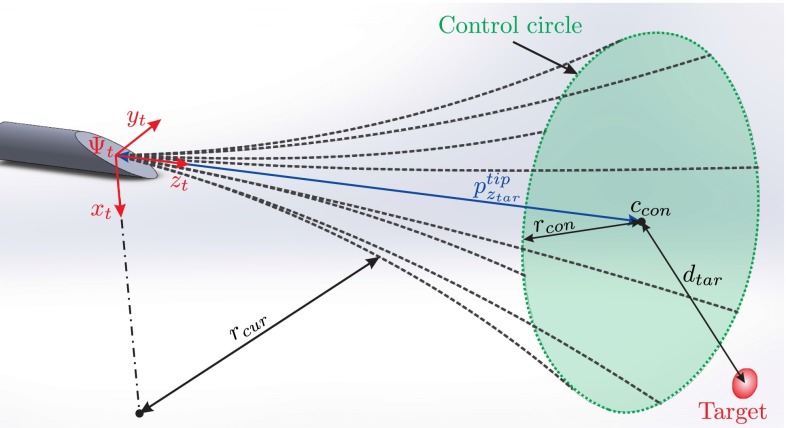
Representative figure explaining the steering algorithm: The needle naturally goes on a *circular* path when inserted in soft tissue. The radius of this *circle* is called radius of curvature ($r_{\text {cur}}$), and the *center* is on the $x_t$ axis. The region the needle tip can reach is a *conical* shape. *Dashed lines* show examples of possible needle paths. The frame ($\varvec{{\varPsi }}_t$) is attached to the needle tip, and the needle is inserted in the $z_t$-direction. The control *circle* with *center* ($c_{\text {con}}$) intersects the target and is perpendicular to the $z_t$-axis. The radius ($r_{\text {con}}$) is determined using ($r_{\text {cur}}$) of the needle and the distance ($p^{\text {tip}}_{z_{\text {tar}}}$ ) between the tip and target along the $z_t$-axis. The needle rotates about its axis to align the tip orientation with the target if the distance between $c_{\text {con}}$ and target ($d_{\text {tar}}$) is larger than or equal to $r_{\text {con}}$

Three steering experiments are performed to prove the functionality of the proposed device. The steering algorithm is based on the method proposed by Abayazid et al. [25]. As discussed earlier, bevel-tipped needles naturally bend when inserted into soft tissue. The direction of the arc depends on the axial orientation of the needle. Dashed lines in Fig. 5 show examples of possible needle paths. These lines form a conical space and define the area which can be reached by the needle. The steering algorithm always keeps the target in this reachable volume by rotating the needle when the target approaches the boundaries of the conical space. This algorithm guarantees the minimum number of needle rotations. This is an important factor due to tissue damage, and subsequent patient trauma caused by other methods such as duty-cycling [10]. The algorithm is represented in Fig. 5 and extensively discussed in our previous work [25].

**Fig. 6:**
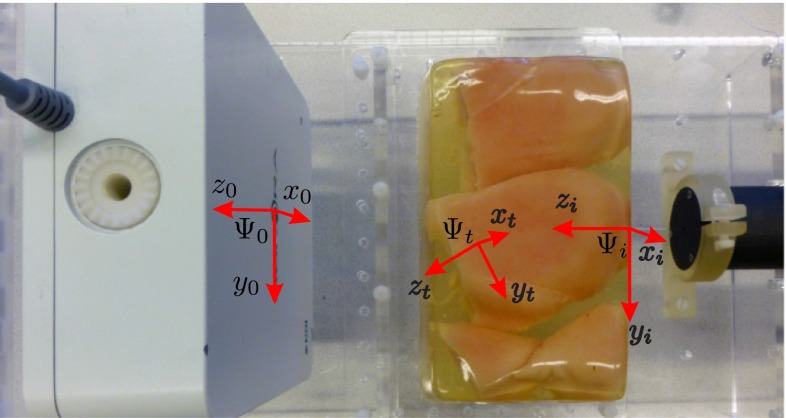
Different coordinate systems required to compute the needle tip pose. Fixed reference frame $\left( \varvec{{\varPsi }}_0\right)$ is located at the *center* of the planar field generator. Frame $\left( \varvec{{\varPsi }}_i\right)$ is at the insertion point on the phantom. Frame $\left( \varvec{{\varPsi }}_t\right)$ is attached to the needle tip

We have used three experimental cases to apply the above steering method. The steering algorithm requires feedback of the needle tip pose, and this is provided using CT images or the EM tracking system. These three experimental cases show how feedback influences the targeting error. In all the cases, the needle is steered toward a virtual target positioned at 6, $-2$ and 90 mm in $x, y$ and $z$ direction, respectively, relative to frame ($\varvec{{\varPsi }}_i$). Please see Fig. 6 for the assigned reference frames. The needle used in the experiments has a diameter of 0.55 mm and has a bevel angle of $30^{\circ }$ at the tip. The insertion speed is 1 mm/s. The phantoms are made by embedding biological tissue (chicken breast) in gelatin in order to fixate the biological tissue. Experimental parameters are the same for all three cases.

*Case 1* In the first case, the needle is steered in an open-loop manner. Steering is performed using only the deflection model of the needle. The control commands are computed based on the simulation using the deflection model. There are uncertainties in the biological tissue properties with respect to a homogeneous gelatin phantom for which the open-loop controller cannot compensate.

*Case 2* In the second case, the needle is steered in a closed-loop manner. Complete needle tip pose is computed online using EM tracker data and the motor encoder. These data are fed back to the steering algorithm, and the result, which is the required amount of needle rotation, is provided to the low-level controller. Due to high refresh rate of the needle tip position in this case, it is possible to compensate for the errors in the system.

*Case 3* In the last case, the needle is steered in an intermittently closed-loop manner. CT images are used as feedback, and therefore, pose data cannot be accessed in real time. A new CT scan is performed after every 20 mm of insertion. The needle tip pose is then extracted from CT images by applying a B-spline interpolation and finding the center of the needle in each image slice [26]. The tip pose is then manually provided to the steering algorithm, and steering is done for the next 20 mm. A final scan is performed after reaching the target depth in order to determine the targeting error.

### Results

**Fig. 7:**
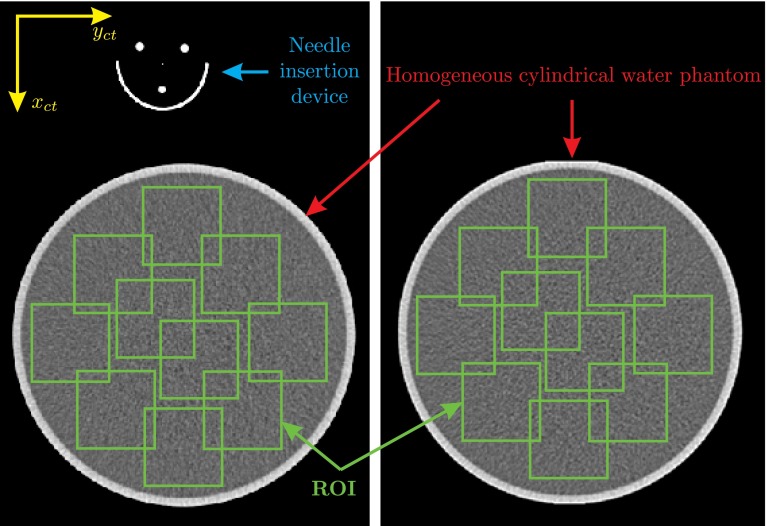
CT noise analysis: The noise power spectrum is computed for a homogeneous cylindrical water phantom. *Left* when the needle insertion device is on top of phantom. *Right* when only the phantom is in the CT scanner. *Green squares* show 10 region of interests (ROI) that are used to compute the noise power spectrum

**Fig. 8:**
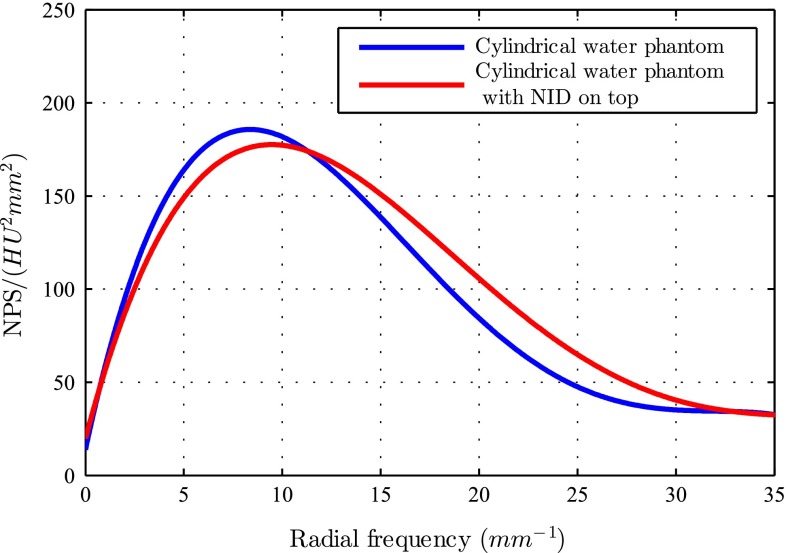
1D (radial) noise power spectrum (NPS): the *red curve* depicts the NPS when the needle insertion device (NID) is on *top* of phantom, while the *blue curve* shows the NPS when the phantom is in the CT scanner alone. HU is the Hounsfield unit

NPS is computed for the case that the water phantom is in the bore alone and also when the NID is placed on top of the phantom. CT scan is performed over the length of the phantom. The NPS is averaged over 10 ROIs in a single image slice. As shown in Fig. 7, no distortion and/or artifacts, due to the presence of the NID in the image plane, are introduced to the images. 1D NPS is presented in Fig. 8 for both experiments. It is shown that the presence of NID does not add a considerable amount of noise to the image. The low-frequency noise is almost the same in both cases, and high-frequency noise is increased about 30 %.

**Fig. 9:**
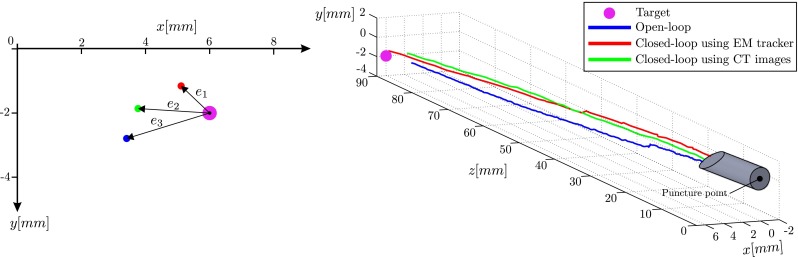
An example of experimental result for each experimental case: Left: the absolute distance between the final needle tip position and target position in $x$-$y$ *plane* is the targeting error. The errors ($e_1, e_2$ and $e_3$) are 0.78, 2.30 and 2.53 mm, respectively Right: the needle is steered toward a virtual target. The needle tip trajectory is demonstrated

For the needle steering experiments, each experimental case is performed 5 times. The results are presented in Table 1 based on targeting error. The targeting error is the absolute distance between the target position and final needle tip position, as depicted in Fig. 9 (left). The experimental case 1 has the largest targeting error. That is due to the fact that the model is based on the assumption that the tissue is homogeneous. However, biological tissue is inhomogeneous which causes changes in the bending radius during the insertion. These uncertainties cannot be compensated for during the open-loop steering. On the other hand, while using online feedback, the steering is updated using the actual pose of the needle tip. This results in minimal targeting accuracy. However, the uncertainties are still in the system and cannot be completely compensated. In case 3, there is intermittent feedback to the control loop. This intermittent feedback results in better targeting accuracy than the open-loop control, but the error is higher with respect to case 2. An example of needle tip trajectory for each experimental case is presented in Fig. 9 (right).

**Table 1:** Targeting error: case 1- open-loop control, case 2- closed-loop control using electromagnetic tracker, case 3- closed-loop control using computed tomography images

Experimental case	Targeting error (mm)	Standard deviation (mm)
Case 1	2.86	1.14
Case 2	1.11	0.14
Case 3	1.94	0.63

## Discussion

In this study, the design and control of a CT-compatible NID are presented. The device is used to steer a bevel-tipped flexible needle toward a virtual target in biological tissue. The NID has two DOF, which are used to insert and rotate the needle. Bevel-tipped needles naturally bend while being inserted in soft tissue. The steering algorithm uses this property, and the needle is controlled to reach the target through a combination of rotations during the insertion. Three experimental cases (open-loop control, closed-loop control using EM tracking system, closed-loop control using CT images) are considered. The average targeting error is $2.86\pm 1.14$ (case 1), $1.11\pm 0.14$ (case 2) and $1.94\pm 0.63\,\hbox {mm}$ (case 3). Open-loop control results in the highest targeting error. This is due to uncertainties in the experimental parameters. The most important source of uncertainty is the radius of curvature. The radius of curvature is estimated for the biological tissue before the experiments. However, this parameter not only depends on environmental parameters (such as temperature), but also depends on the phantom location where the needle is inserted. Tissue layers in the phantom can cause the needle to deflect in a different direction than expected. Since there is no feedback from the needle pose in the open-loop case, the mentioned errors are not detected and hence are not compensated for by the control algorithm. On the other hand, closed-loop control using EM tracking has the least amount of error. Due to the high refresh rate and online feedback of the tip position to the control algorithm, it is possible to compensate for the errors. In case 3, the pose of the needle tip is computed 5 times during the complete insertion. This case is similar to the current clinical procedure in which clinicians take several CT scans during the procedure. Using this intermittent feedback, it is possible to close the control loop and achieve better targeting accuracy than open-loop control (Fig. 8).

In this study, needle steering was performed in gelatin phantom with biological tissue. In a real clinical lung biopsy procedure, there are also other variables which may influence the targeting accuracy. Target motion due to respiration, various tissue layers with different physical properties and tissue inhomogeneity are among the most important parameters. As a result, further improvements are required to bring the system to the clinical practice. The NID can be extended to provide more DOF around the puncture point. This helps us to modify the initial needle insertion angle. An online path planner is also beneficial to avoid hitting sensitive organs and bony structures [25]. The future work is focused on experiments with moving real targets in biological tissue, which will result in a system that can be used in clinical operations and more specifically lung biopsies.
